# Wild mockingbirds distinguish among familiar humans

**DOI:** 10.1038/s41598-023-36225-x

**Published:** 2023-06-24

**Authors:** Douglas J. Levey, John R. Poulsen, Andrew P. Schaeffer, Michelle E. Deochand, Jessica A. Oswald, Scott K. Robinson, Gustavo A. Londoño

**Affiliations:** 1 Division of Environmental Biology, National Science Foundation, 2415 Eisenhower Ave, Alexandria, VA 22314 USA; 2 Nicholas School of the Environment, Duke University, Durham, NC 27708 USA; 3 Florida Museum of Natural History, University of Florida, Gainesville, FL 32611 USA; 4 Department of Biology, University of Nevada, Reno, NV 89557 USA; 5 Departamento de Ciencias Biológicas, Universidad Icesi, Cali, Colombia

**Keywords:** Urban ecology, Behavioural ecology

## Abstract

Although individuals of some species appear able to distinguish among individuals of a second species, an alternative explanation is that individuals of the first species may simply be distinguishing between familiar and unfamiliar individuals of the second species. In that case, they would not be learning unique characteristics of any given heterospecific, as commonly assumed. Here we show that female Northern Mockingbirds (*Mimus polyglottos*) can quickly learn to distinguish among different familiar humans, flushing sooner from their nest when approached by people who pose increasingly greater threats. These results demonstrate that a common small songbird has surprising cognitive abilities, which likely facilitated its widespread success in human-dominated habitats. More generally, urban wildlife may be more perceptive of differences among humans than previously imagined.

## Introduction

A wide variety of arthropods and vertebrates can distinguish among individuals of their own species^1–6^. This ability is not surprising, as it facilitates mate selection, parental care, communication, and practically all other social interactions. Much less expected and explored is the emerging generalization that individuals of one species can distinguish among individuals of a different species. This ability is most apparent in domestic species such as farm animals and household pets^7–11^ and in vertebrates considered to have high cognitive function, such as corvids, primates and elephants^12–14^. Although most commonly studied with captive animals in highly controlled settings e.g., Refs.^15,16^, wild individuals in natural environments also appear able to distinguish among heterospecific individuals^17–24^. Yet, establishing the occurrence of “true” individual recognition is controversial, even among individuals of the same species^25,26^. At issue is whether the perceiving individual learns unique characteristics of the detected individual or simply distinguishes between familiar and unfamiliar individuals or groups of individuals. The challenge is especially great for documenting heterospecific individual recognition in natural settings.

**Figure 1:**
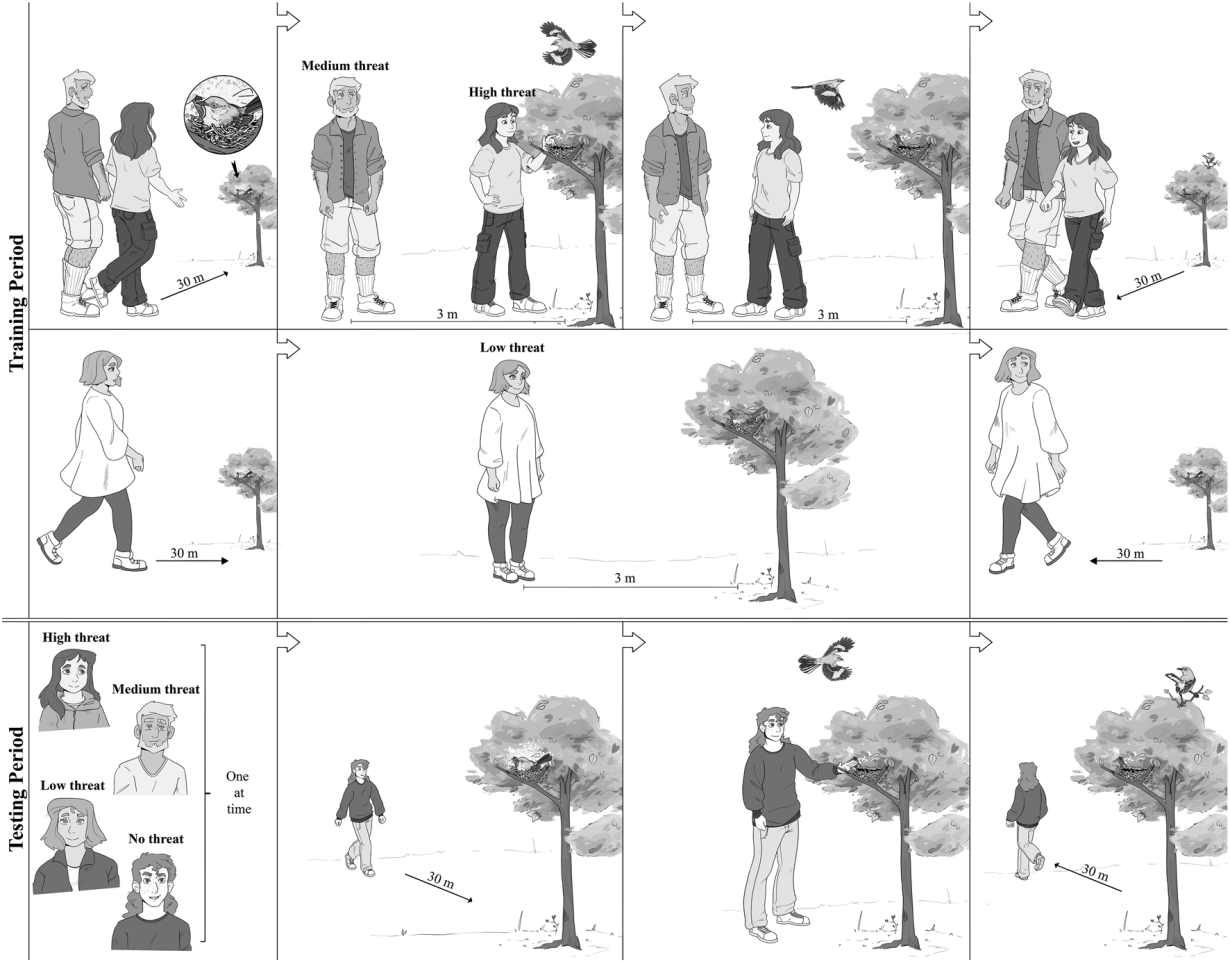
Experimental protocol. Training period, top row: High and Medium Threat individuals approached nest together. The Medium Threat individual stopped 3 m from nest, while the High Threat individual stood at nest for 15 s, placed hand on rim of nest for 15 s, and retreated to where the Medium Threat individual was standing. They faced each other for 15 s before retreating together along the same path. Training period, second row: Low Threat individual approached the nest along a different path and stood 3 m away for 10 min before retreating. The training period lasted 3 days, with one visit of each visitor type per day. Testing period, bottom row: High, Medium, Low and No (control) Threat individuals approached the nest a single time, separately, in random order, with at least 6 h between visits, over a 2-day period. In all approaches, we recorded the distance between the individual and the nest when the bird flushed (Flush Distance). Illustration: José Alejandro Riascos Ramírez.

Here we describe an experimental study with Northern Mockingbirds (*Mimus polyglottos*), a common species in urban environments throughout the continental United States. We systematically exposed free-living birds to human intruders that varied in their degree of threat (Fig. 1). The goal was to test whether mockingbirds could distinguish among them, responding in a manner reflecting the degree of risk. We predicted that female mockingbirds would flush from their nest progressively sooner when approached by familiar humans who posed increasing levels of threat (No Threat, Low Threat, Medium Threat, and High Threat). Such a result would demonstrate the birds’ ability to learn unique cues of each human, to quickly process that information, and to respond in a manner specific and functionally relevant to a wide variety of familiar humans. It would reveal a new level of cognitive function in a small songbird (Passeriformes) and help explain mockingbirds’ ability to thrive in urban environments.

## Results

**Figure 2:**
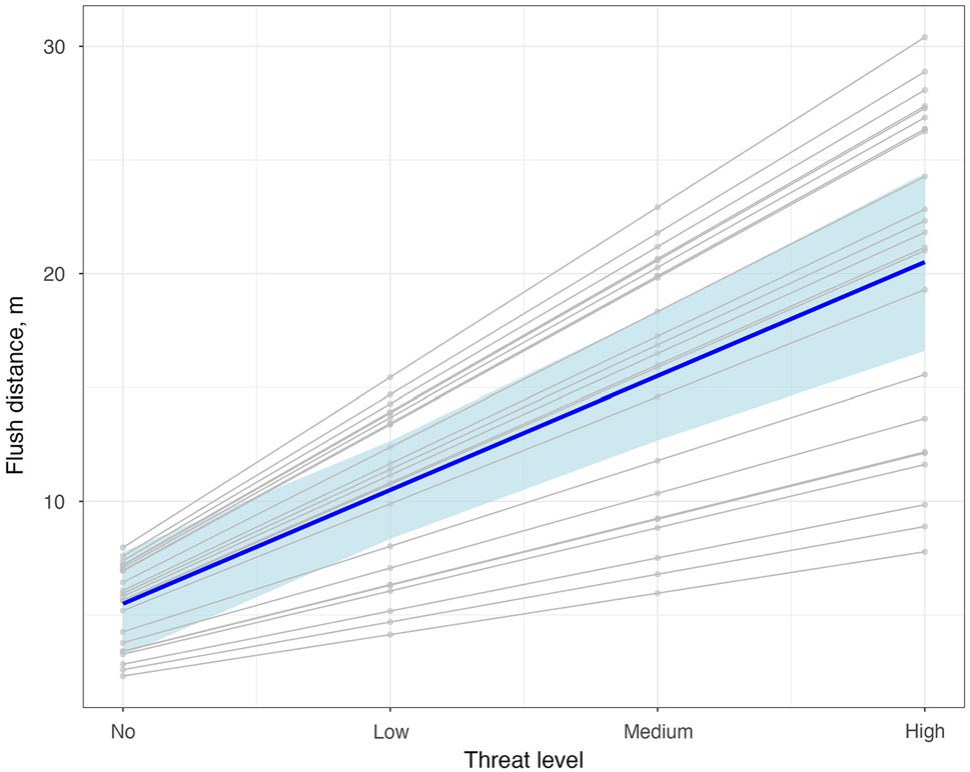
Incubating mockingbirds flush from their nest at greater distances when approached by familiar humans known by them to be more threatening. Blue line shows the average response and shading shows the 95% confidence intervals around the average. Gray lines show model fit for individual birds.

Experimental trials consisted of a 3-day training period, during which incubating females were exposed daily to one human of each threat category except for No Threat, and a 2-day testing period, during which we recorded their responses to a final approach of each human. Over the training period, flush distance increased ~ 2.5 m/day for High and Medium Threat individuals (LMM: t = 3.27, df = 72, p = 0.002) and birds did not noticeably respond to Low Threat individuals, demonstrating the effectiveness of those treatments. On test days, birds responded differently to approaches by the four humans; flush distance increased ~ 5 m for each increase in threat level (LMM: z = 7.10, p < 0.001, Fig. 2 & Supplementary Fig. S1). A varying slope model best fit the data, indicating high variation among individuals in sensitivity to humans of progressively higher threat levels. The model’s total explanatory power was high (conditional *R*^2^ = 0.61), with Threat Level alone explaining 31% of the variance (marginal R^2^). The number of attacks was unrelated to Threat Level (Poisson GLMM, varying intercept model: z = − 1.77, p = 0.077), with Threat Level alone explaining a very small extent of variance (marginal R^2^ = 0.005). Likewise, the number of alarm calls was not related to Threat Level (varying slope and intercept model; z = 1.54, p = 0.12).

## Discussion

These results demonstrate that mockingbirds can recognize multiple familiar humans individually. Prior studies on wild birds have been unable to rule out the possibility that study species were instead making coarse-level distinctions between familiar and unfamiliar humans^18–23,27–29^. The flush distances of mockingbirds were graded, proportional to the level of threat posed by each individual who approached the nest. This individual-specific receiver response requires the ability to distinguish among multiple types of heterospecific individuals and characterizes the strictest definition of individual recognition within species^1,25,26^; it has not previously been documented between species, except for a study involving only two types of familiar humans^17^. Remarkably, mockingbirds learned to identify humans and assess their threat levels in as little as three exposures of 30 s near the nest.

Understanding animals’ ability to distinguish among individuals of a different species is important for two reasons. First, it reflects cognitive ability because it requires integration of learning, recalling, and applying information^30^. Those processes are well-established in parrots and corvids, which are considered cognitively superior to other birds^31–33^. Other avian taxa remain largely overlooked and untested in ecologically relevant settings^19,23^. Our results add to the growing consensus that cognitive function is likely greater and more phylogenetically widespread in birds than generally recognized^34,35^.

Second, because practically all studies on individual recognition of heterospecifics have tested the ability of various animals to distinguish among people, results have direct relevance to human-wildlife interactions and urban ecology^36,37^. Specifically, they reveal the extent to which species become aware of and can respond to differences in human behavior. Given that humans are highly variable in their interactions with wildlife, the ability to distinguish quickly among individuals who pose different degrees of risk or gain would be highly beneficial and could partially explain which species are most likely to thrive in human-dominated environments^38–43^, but see Ref.^23^.

In addition to mockingbirds’ surprising cognitive function, another factor may facilitate their ability to learn quickly to discriminate among familiar humans^22^: consistent exposure to hundreds of non-threatening people could help them learn which human stimuli are consistently distinctive (e.g., facial features) and which are not (e.g., clothing)^17^. Then, when a particular human becomes a threat or benefactor, the birds are more able to quickly learn the person’s unique characteristics than if they had not previously watched so many humans.

We conclude that nesting mockingbirds are keenly aware of nearby humans as individuals, not just collectively. Daily, hundreds of students walked past the nests in this study and the birds ignored the vast majority. Yet, they responded quickly to a few individuals in a manner proportional to those individuals’ unique level of threat. If the underlying cognitive processes of this behavior occur in other taxa, individual differences in human behavior may play a large role in structuring communities of urban wildlife.

## Methods

Our study took place in Gainesville, Florida on the ~ 800-ha campus of the University of Florida. Northern mockingbirds were chosen for study because they are common on campus and urban areas, frequently nest in isolated shrubs or small trees, and are known to distinguish between familiar and unfamiliar humans^24^. Furthermore, they are highly acclimated to the presence of humans, typically showing no response unless approached within 2–3 m. The average number of pedestrians passing within 5 m of nests over a 5 min period is 4.5 ± 6.4^24^. Pairs are socially monogamous and territorial. Territory size is 1–2 ha on campus. Only the female incubates^44^, which allowed us to tightly control a given bird’s exposure to different humans. Because much of the population was individually color banded and the study occurred during a single breeding season on widely separated territories, we are confident that each female was included only once in our sample.

When the nest of an incubating female was discovered, we first established three 30 m paths along which an approaching human could be seen from the nest. In some cases, a person not participating in the trials removed a small number of leaves or twigs near the nest to assure equal visibility of approaching humans along all paths. The purpose of multiple approach paths was to minimize the possibility that the bird might respond to the direction of approach rather than (or in addition to) the identity of the human. For each nest, assignment of High, Medium, Low and No Threat individuals was randomized. Approach paths were randomly assigned, then sequentially rotated so that threat level could not be predicted by path location. The rate at which all individuals approached and retreated from nests was calibrated and standardized. We tested 24 incubating females during peak nesting period (May and June) in 2009. Trials on a given nest occurred over five consecutive days (2 trials per day), divided into a training period of 3 days and a testing period of 2 days.

*Training period* On the first 3 days of a trial, the nest was visited by a pair of High and Medium Threat individuals simultaneously, and at least 3 h before or after a visit by the Low Threat individual. The goal was to establish the three treatments—i.e., to familiarize the female with each type of human and their threat level. Prior work using a similar protocol established that mockingbirds significantly increase their response to threatening humans after 2 visits to a nest^24^. High Threat individuals approached the incubating female from 30 m at a rate of 1 m/s, maintaining eye contact with her, until they were ~ 1 m from the nest or she had flushed from the nest. They stood still for 15 s without making eye contact with her and then placed one hand on the rim of the nest for 15 s before retreating. The Medium Threat individual walked beside the High Threat individual but stopped 3 m from the nest and stood still, looking towards the nest while the High Threat individual proceeded to it. When the female flushed, neither individual looked at or responded to it. After leaving the nest, the High Threat individual walked back ~ 2 m and stood facing the Medium Threat individual within ~ 0.5 m for 15 s before both traveled together along the same path and at the same rate as they had approached. Low Threat individuals approached the incubating female along a different path at 1 m/s while maintaining eye contact with her, stopped 3 m away, stood quietly for 10 min without looking at her or the nest, and then retreated in the same manner as High Threat and Medium Threat individuals (except for path). The No Threat individual (Control) did not approach the nest during the training period, only once during the testing period. We recorded the approaching human’s distance from the nest when the female left the nest (Flush Distance), and the number of alarm calls and attacks. Alarm calls are distinctive—loud, of short duration and with a wide bandwidth. Attacks were defined as downward flights within 1 m of the intruder’s head. In rare cases, they resulted in direct contact (usually with the bird’s feet).

*Testing period* On the last 2 days of a trial, each incubating female was exposed to one individual of each threat level, including the No Threat individual who had not previously approached the nest. The order and paths of these approaches were randomized. Two trials occurred per day, separated by > 6 h. In these four trials, all humans behaved identically, except for the approach path used. They approached the nest from 30 m at a rate of 1 m/s, paused ~ 1 m from the nest for 15 s, touched the rim of the nest for 15 s, and walked away at the same rate and along the same path as they had arrived. None paused 3 m from the nest. We collected the same data as during the training period.

We emphasize that our experimental design did not employ distinctive clothing or masks with exaggerated features that the birds could associate with treatments. All participants wore what they normally would have worn if they had not been participating in a trial. The only restriction was that they could not wear hats or sunglasses. Except for the first author, all participants in trials were university students, presumably indistinguishable at the start of trials from the other ~ 50,000 students on campus at the time. Our goal was to mimic as closely as possible the natural variety of humans near each bird’s nest. Nine women and five men participated in the trials, all varying in complexion and stature.

Because all trials were conducted during the focal females’ incubation period, our experimental design also controlled for the well-known association between nest defense behavior and stage of the breeding cycle^45^. If any eggs hatched or disappeared, we discontinued trials on the impacted nest.

All methods were carried out in accordance with relevant guidelines and regulations. Specifically, we adhered to an experimental protocol that was approved by the University of Florida Institutional Animal Care and Use Committee (protocol #D884-007) and that followed ARRIVE guidelines.

### Statistical analyses

Using mixed models (LMM, GLMM) with the appropriate probability distribution, we assessed the effects of threat level, including it as a covariate, and bird individual, treating it as a random effect to account for repeated measures. We treated the threat levels as continuous and assumed they increased linearly from No Threat to High Threat. We compared three models with different random effect terms: (1) a varying intercept model with location as a random effect to account for individual variation in average level of response; (2) a varying slope model with visit as a random effect to account for individual variation in the response across threat levels; and (3) a varying intercept, varying slope model that accounted for both types of variation in response. We used the Likelihood Ratio Test to compare models.

For flush distance, a continuous response, we used linear mixed models (LMM). We evaluated the assumptions of homoscedasticity and normality of residuals by visually examining residual plots and QQ-plots of the residuals. For number of attacks and alarm calls, we used generalized linear mixed models (GLMM) with a Poisson distribution and log-link. We checked the assumption that the variance is equal to the mean (lack of overdispersion) and compared the predicted to observed counts through rootograms. Mixed models were run in the *glmmTMB* (version 1.1-28) package, and all statistical analysis were conducted in R version 4.1.2. We calculated a pseudo-R2 for mixed models using the trigamma function in the *MuMIn* package.

## Supplementary Information

Supplementary Figure S1.

## Data Availability

The datasets generated during the current study are available in the Dryad repository, https://doi.org/10.5061/dryad.p2ngf1vvq.
