# Supplementary material for: Wild mockingbirds distinguish among familiar humans
Source: Sci Rep. 2023 Jun 24;13:10259. doi: 10.1038/s41598-023-36225-x (PMC10290633; doi:10.1038/s41598-023-36225-x)
Supplement: Supplementary file 1 — Supplementary Figure S1. [file 41598_2023_36225_MOESM1_ESM.pdf]

Supplementary Figure S1

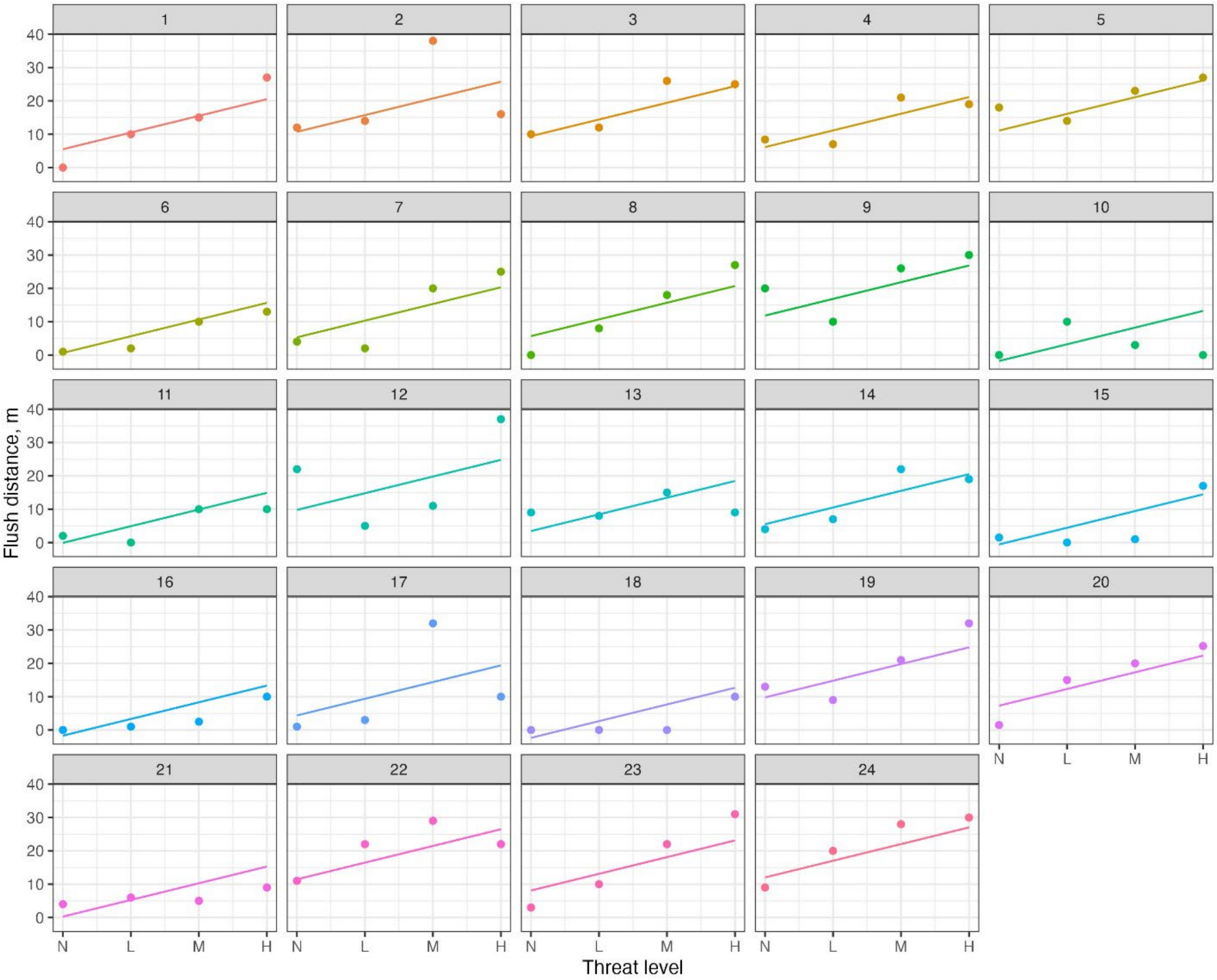

Flush distance from nest as a function of threat level of humans who approach the nest. N = No Threat (control); L = Low Threat; M = Medium Threat; H = High Threat. Numbers above each graph identify the 24 different females tested.
